# Monthly Review of Parisian Medicine and Surgery

**Published:** 1859-10

**Authors:** Theophilus Mack

**Affiliations:** Lecturer on Materia Medica and Therapeutics in the Medical Department of the University of Buffalo


ART. II.—*Monthly Review of Parisian Medicine and Surgery:* By THEOPHILUS MACK, M. D., Lecturer on Materia Medica and Therapeutics in the Medical Department of the University of Buffalo.

OUR monthly mirror refused to reflect last month, for lack of French polish, the supplies having unexpectedly intermitted, from the delinquency of agents. We shall put forth extraordinary efforts, in our bee-like capacity, of extracting French bon-bons, if our *caro* editor will only not turn waspish on our shortcomings, and our *piu caro publico* will only blame the true drones—*the agents.*

Dr. Béclère communicates the history of a new case of croup, cured by T. emetic in large doses; another case to swell the number of observations published by M. Bouchut. The case was of the gravest description. After a cough of three days' duration, the following symptoms were presented: "Fauces pale, discolored; general anxiety; parotid glands engorged, especially the left; mucous discharge from nasal fossæ; respiration hoarse and loud, with intense wheezing or hissing at the highest efforts for respiration which the unfortunate child would make, and which were provoked every moment by suffocation; in a word, asphyxia appeared imminent. Upon depressing strongly the tongue, false membranes might be discerned on the back part of the throat, and the laryngeal mucous membrane; farther, there existed a general obstruction of the lungs. The child had coughed a good deal in the evening, and its cough had a deep and hoarse sound; the cough had ceased since morning, and at the same time the voice became extinct." The child was aged four years and seven months; and M. Béclère made the prescription of tartarized antimony in gum potion (120 grammes of this vehicle containing 0.75 centigramme of T. emetic); of this, half a tablespoonful every half-hour. The improvement commenced at once, and on the second day chlor. pot. was prescribed. Convalescence ensued.

Among many invaluable observations by that zealous medical philosopher, M. Claude Bernard, we notice in his "Lessons upon the Physiological Properties and Pathological Alterations of the Liquids of the Organism," the following respecting the temperature at different points in the course of the blood.

The venous blood in the cava and neighboring veins, compared to the blood at the arch of the aorta and its direct branches, has constantly an inferior temperature.

The blood of the renal vein is warmer than that of the emulgent artery.

The portal blood is cooler than that of the inferior hepatic veins, and more warm than that of the descending aorta, immediately above the diaphragm.

The blood of the veins of the lower extremities is less heated than that of the corresponding arteries; it is the same with the blood of the iliac arteries and veins. The blood of the ascending cava, as far as the opening of the renal vein, is also of a lower temperature than that of the descending aorta below the origin of the renal arteries.

The mixture of the blood of the renal vein with that returned from the lower limbs, produces this result: that in all the portion of the vena cava comprised between the opening of the renal veins and the liver, the blood is warmer than in the portions of the abdominal aorta extending from the diaphragm to the origin of the renal arteries.

"At the moment when the infra-hepatic veins disgorge themselves into the vena cava ascendens at the temp. of 39.80 cent., the heat of the blood of this last vein is elevated from 39.40 to 39.65, and carries it a good deal higher than that of the blood of the corresponding part of the aorta (38.70). The confluence of the hepatic vein with the cava is the warmest place in the economy, 39.80. In the right auricle, the very warm blood of the inferior cava (39.50 to 39.65) mingles with the blood of the superior cava (39.20); its temperature falls (to about 39.35) below what it was at the base of the diaphragm (39.50), but still remaining superior to that of the blood of the descending aorta (38.70). Always, the blood of the right ventricle of the heart (39.32), with living animals, is warmer than the blood of the left ventricle (39.07), giving in favor of the right ventricle 0°.25."

"The Society of Biology," in their last volume of Transactions, publish two memoranda by M. Bernard; the first entitled: "New Experimental Researches upon the *glycogenic* phenomena of the Liver;" the second: "Notes upon the variable quantities of Electricity necessary to excite the Properties of the Various Tissues." To the first of these we have already directed attention in a former "Review;" with regard to the second: M. Cl. Bernard, serving himself with the instrument contrived by M. Jules Regnault, for the estimation of the quantity of electricity employed in his experiments, has shown that a more energetic current is required to make the salivary gland produce its usual secretion by the irritation of the chorda tympani, than is required to cause contraction of a muscle by the irritation of the facial nerves. He has also shown this fact: that to produce contraction of the pupils, or the blood vessels, by irritating the cervical twig of the great sympathetic, a much greater quantity of electricity is required, than to excite a nerve of animal life. The vital properties of the motor nerves require a difference of quantity of electricity from the muscles, for their excitation; that exciting the trunks determines contractions more readily than excitement of the muscles themselves. A difference of excitability also is perceptible between a motor and a sensitive nerve.

It would appear that the nerve of Wusberg is not, as generally received, a sensitive root constituting with the facial nerve a complete nervous pair; but is very probably a radicle of the great sympathetic, with which it appears to partake in the influence upon the glandular secretions. It is also apparent, that the submaxillary glands of each side receive sympathetic nerves from two sources,—the one accompanying the nerve of taste, and furnished by the cord of the tympanum; the others ascending from the intra-abdominal ganglia and plexuses towards the superior cervical ganglion, to terminate in the glands; that the filaments emanating from the chorda tympani act in a centrifugal sense, in the manner of the motor nerves; and that under the influence of their section, the salivary secretion is interrupted.

A contribution from Messrs. Rousseau Lesure and Martin-Magron, entitled "Action of Electric Currents studied comparatively upon the mixed nerves and upon the anterior spinal roots," appears to us of much importance. In this work is rectified an error of Matteuci, viz., "that a galvanic current, which traverses a portion of the length of a nerve, acts in the same manner upon the isolated movements of the muscles to which this nerve is distributed, such nerve be it mixed (that is to say, at the same time sensitive and motor, as the sciatic nerve), or be it exclusively motor (as an anterior spinal root)." The facts are—

1st. In the greater part of the experiments, where a galvanic current is made to act upon a nerve dissected out, there is established a derived current, easily demonstrable, which often gives results completely opposed to those which the principal current furnishes when it exists alone.

2d. Of two opposite currents acting simultaneously at a different height upon the same nerve, that which is nearer the periphery manifests its action only by contractions in the muscles animated by this nerve; it opposes itself as a barrier to the transmission of the nervous action developed higher up by the current in an opposite direction.

Hence, a mixed nerve and an exclusively motor nerve, both react in the same way respecting the contraction peculiar to the muscles they animate, when they are submitted, in the same conditions, to the influence of a galvanic current in the same direction. Thus, no matter what may be the nature of the nerve, in a first period, the direct and the inverse current both determine contractions at their establishment and at their rupture; then, at a second period, the nerve having lost its excitability, the contractions only take place—1st. At the establishment of the direct current; 2d. At the rupture of the inverse current.

We must also mention a note by M. Felix Garmal upon hydropisine, a new albuminoid matter, confounded up to this day with albumen.

At "The Academy of Sciences," M. Flourens presented, in the name of M. Paolini, Professor of Physiology in the University of Bologna, a memoir relative to new observations made by the author upon the spinal chord; observations which are thus summed up:

1st. The posterior and lateral columns of the spinal marrow are endowed with exquisite sensibility.

2d. The division of these cords does not hinder the transmission to the encephalon of sensitive impressions.

3d. The impressions transmitted by the posterior spinal roots, after a short passage across the medullary fibres of these cords, pass into the gray substance.

4th. The gray substance, although insensible itself, that is to say, incapable to receive direct impressions of excitement of feeling, appears to be the indispensable medium for the transport of these impressions to the sensorium communum.

5th. The posterior branches alone being cut transversely, the sensibility of the parts of the animal situated below the section augments temporarily.

6th. The posterior branches preserve their own proper sensibility, even when divided at two or three points, a certain distance one from the other.

7th. The anterior branches are insensible to the direct application of stimuli.

8th. Lastly, these anterior branches are essentially motor, but seem to be no strangers to the production of feeling.

Gentlemen indulging in the fancy for Shanghais and Dorkings will be pleased to learn that—1. Hens are liable to a skin disease depending on the *acarus mutans*; 2. The resulting trouble resembles the itch in the genus homo; 3. It is transmitted from one fowl to another; and, 4. It is also transmissible to animals.

Several deaths are noticed as having resulted from the heat in the southern departments. The brain was found in all cases in a state of extreme congestion. Cholera infantum is also mentioned as having made its appearance. What a happy land to open the morning of one's days in, where cholera of infants occurring during the dog-days is a matter worthy of note!

M. Ollier has ascertained that the dura mater, in young animals especially, has the property of secreting bone. Flaps of dura mater engrafted in the axilla and groins of animals, gave rise to small and well-formed bones, with all the anatomical characters of normal osseus substance.

Dr. Wood's method of injecting narcotics has found grace in Parisian eyes. Atropine and strychnine and muriate of morphia are used; and it is stated that pain was always relieved, and cures were effected in all the cases where the injections were sufficiently repeated. We have not found these subcutaneous injections answer quite the expectations raised by the periodicals, and we think the statements must be taken "*cum grano salis.*"

M. Bourdon presented to the "Medical Society of the Hospitals" a biliary calculus of considerable volume, which, after falling into the digestive-tube through the adherent and perforated walls of the transverse colon and gall-bladder, was arrested at the sigmoid flexure, where it caused fatal lesion.

The black-cancer Hercules has at last been stripped of his pseudo-honors, by the death of the credulous victim of his charlatanry, Mad. de Bougemont. Will it be believed that before setting out for the army of Italy, the Emperor sent for Vriès, and expressed himself so confident in the impostor that he delayed his departure in order to follow up his prescriptions? It is to be regretted that M. Velpeau should have allowed *La Charité* to become the theater for attracting public attention to this pretender.

M. Conturier publishes a successful case of strangulated hernia treated by infusion of coffee (lb. ss. freshly ground, to boiling water O iij.).

Dr. Laforgue has succeeded in reducing strangulated hernia, by placing the patient's head downwards, almost vertical, and kneading the abdomen.

Attention is drawn in the "*Bulletin de Thérapeutique*" to the use of the pulp of raw meat in infantile diarrhœa. This is a remedy we have now tested fully for about eight years, and we can speak of it in the highest terms of praise. Raw mutton or beef should be minced, pounded, and then strained. The red fluid thus obtained is to be incorporated with jam or sugar, and administered ad libitum.

M. Beau has brought up the treatment of phthisis by white lead, based upon five successful cases. Two grains, gradually increased to sixteen, are given each day until saturnine poisoning begins to set in.

A disinfectant, recommended by Drs. Demraux and Corne, is made as follows. Plaster of Paris, 100 pts., finely powdered; coal-tar, from 1 to 3 pts.; mix in a mortar; add a sufficient quantity of olive oil to reduce the mixture to the consistence of ointment, and preserve for use in a close vessel. M. Velpeau has confidence in this invention. The advantages it offers are thus summed up:

1. A gangrenous wound, emitting fœtid and abundant pus, is at once deprived of its bad smell.
2. After a twenty-four or thirty-six hours' application, the bandages of a bad sore exhale no more smell than if they had been applied to a common fracture.
3. A cancerous ulcer is immediately deprived of its fœtor.
4. The same is the case with ulcers of the legs.

5. Bandages and poultices charged with offensive pus, are at once disinfected, when brought into contact with the compound above described.

6. It also stops decomposition, keeps away insects, and prevents the generation of worms.

An important article upon one of the sequelæ of diphtheria, paralysis of the larynx, has been contributed by M. Maingault. General paralysis also follows, sometimes. Recovery is very general, and may be promoted by all the usual cerebro-spinal excitants, and by steel, sulphurous baths, saline baths, douches, and vegetable tonics.

M. Verneuil submitted a patient for examination, to the "Chirurgical Society," who had undergone "resection of the elbow, with preservation of the periosteum," or sub-periosteal re-section, on the 29th January, or about 100 days previously. According to this method, eight centimetres of the humerus and three centimetres of the two bones of the fore-arm were removed. Cicatrization advanced very quickly, having been only slightly retarded by the consecutive discharge of two little splinters. The cicatrices on the day of presentation were solid, and the site of the operation is not the seat of any pain; the extremities of the resected bone are solidly reunited by strong fibrous tissue, permitting very great mobility between the arm and fore-arm. Well-marked enlargements are visible at the extremities of the bones, very favorable to the strength and precision of the movements. This happy result is clearly ascribable to the preservation of the periosteum.

M. Démarquay reports a case of fistula, the result of fracture of the frontal sinus, communicating with the nose, and treated successfully by autoplasty *par glissement*. "About two centimetres above the root of the nose, and nearly in the median line, was perceived a triangular cicatrix, nearly three centimetres in height, and two in width. At its center was a hole almost triangular, admitting the extremity of the little finger. This hole, by an opening obliquely downwards and backwards, gives access to the frontal sinus. A stylet introduced penetrates into both the nasal fossæ. All around is seen a depressed cicatrix of a pale rose-color, adhering to the circumference of the bony opening. This hole does not give passage to any oozing; but upon making the patient blow, with the nostrils shut, the nasal mucus is seen passing through it."

*The operation.* "The cicatrized tissue having been circumscribed by the cutting instrument, and raised by dissection, the operator made a reversed T incision; the horizontal branch extended from one eyebrow to the other, passing just above the root of the nose; the vertical incision fell perpendicularly upon the middle of this incision; this followed nearly the median line of the forehead, and ended a little in front of the hair. He thus obtained two angular strips; each of these was detached by dissection to an extent sufficient to permit easily the approaching of the edges of the vertical incision. The bleeding surfaces being cleaned, and a great number of small arteries tied carefully, M. Démarquay employed the twisted suture throughout the extent of the incisions. Compresses, wet with cold water, were then permanently applied upon the frontal region."

The next day the state of the patient was very satisfactory; the inflammatory reaction had been very moderate. The pins were withdrawn on the fifth day, and replaced by strips of diachylon. Eleven days after the operation the patient quitted Paris, reunion being complete, and the cicatrix solid and permanent.

M. Boucher treats fissure of the anus by introducing into the rectum the index finger of each hand, and dilating with considerable force until all resistance is overcome.

M. Chassaignac removed a portion of the tongue by the ecraseur, for cancer. The chain was tightened to the extent of a link every half-minute.

In subcutaneous whitlow of the index finger, M. Boucher states "that inflammation and its consequences do not extend into the palm of the hand, by reason of the attachment of the skin opposite the metacarpo-phalangeal articulation to the deeper structures, thus forming a natural barrier. Swelling and effusion soon appear on the dorsum of the hand, because, in this region, the fibrous barrier is absent. Again, inflammation and abscess in the tendinous sheath of the fingers do not pass into the palm, because the tendinous sheath ceases at the metacarpo-phalangeal articulation; but in the thumb, the sheath is continued into the hand; therefore pus may be found in the palm. Also when the interior of the sheath is affected, the finger will be more or less flexed."

